# Postprandial plasma amino acid, glucose, and insulin responses in healthy dogs following the consumption of extruded diets containing cricket powder or poultry meal

**DOI:** 10.1093/jas/skag162

**Published:** 2026-05-22

**Authors:** Larissa Alves Koulicoff, Guanchen Liu, Youhan Chen, Tucker Graff, Reshma Moolakkal Antony, Evan C Titgemeyer, Sajid Alavi, Nick Serao, Júlia Guazzelli Pezzali

**Affiliations:** Department of Grain and Food Science, Kansas State University, Manhattan, Kansas 66506, United States; Department of Grain and Food Science, Kansas State University, Manhattan, Kansas 66506, United States; Department of Grain and Food Science, Kansas State University, Manhattan, Kansas 66506, United States; Department of Grain and Food Science, Kansas State University, Manhattan, Kansas 66506, United States; Department of Animal Sciences and Industry, Kansas State University, Manhattan, Kansas 66506, United States; Department of Animal Sciences and Industry, Kansas State University, Manhattan, Kansas 66506, United States; Department of Grain and Food Science, Kansas State University, Manhattan, Kansas 66506, United States; Statsgaze Data Science Solutions, Rio de Janeiro 20000-000, Brazil; Department of Grain and Food Science, Kansas State University, Manhattan, Kansas 66506, United States

**Keywords:** canine, carbohydrate, insect, kinetics, rendered ingredient, protein

## Abstract

Crickets are emerging as a high-quality, sustainable protein alternative to traditional animal-based ingredients in canine nutrition. Although some studies have evaluated the apparent total tract digestibility of macronutrients in canine diets containing cricket powder (CKP), data on the postprandial metabolic effects of CKP are lacking. This study evaluated postprandial plasma glucose, insulin, and amino acid (AA) responses of healthy adult beagles fed extruded diets containing 30% poultry meal (CTRL) or CKP (CKP-D). Eight dogs were enrolled in a crossover design consisting of two experimental periods of 7-d each. On the final day of each period, dogs underwent a postprandial meal response test with serial blood sampling to assess plasma glucose, insulin, and AA kinetics. Data were analyzed using the GLIMMIX procedure in SAS Studio (v3.81) with diet, time, and their interaction as fixed effects in a repeated-measures model. A time effect (*P *< 0.05) was observed for glucose and insulin, with both peaking from 60 to 180 min following meal consumption. Except for maximum glucose concentrations, which were greater (*P *< 0.05) in dogs fed CKP-D, no differences (*P *> 0.05) between diets were detected for other glucose or insulin parameters. Concentrations of all AA, except Gln, were influenced (*P *< 0.05) by the consumption of a meal. Dogs consuming CKP-D had greater (*P *< 0.05) plasma concentrations of most AA, including Arg, His, Ile, Leu, Lys, Met, Thr, and Val. Moreover, incremental areas under the curve (iAUC) were greater (*P *< 0.05) for Ile, Leu, Val, and the sum of branched-chain AA in dogs fed CKP-D, whereas iAUC for Gly was greater (*P *< 0.05) for CTRL. Overall, AA in the CKP-D appeared to have greater postprandial plasma bioavailability than that in CTRL. Further research is warranted to investigate the mechanisms underlying these responses.

## Teaser

Cricket powder is a relatively novel ingredient in pet food, and little is known about its metabolic effects when fed to dogs. This study evaluated the postprandial amino acid and carbohydrate responses in healthy dogs fed an extruded dog diet containing 30% cricket powder compared with a control diet containing poultry meal as the major protein source.

## Introduction

Cricket powder (CKP), also commonly referred to as cricket flour, is a rich source of protein with a favorable amino acid (AA) profile for adult dogs (Valdés et al. 2022). Typical crude protein (CP) content in CKP ranges from 58% to 78% on a dry matter (DM) basis, and the variation in nutrient composition is attributed to the cricket species used for production (eg *Acheta domesticus*, *Anabrus simplex*, *Gryllus spp.*, *Gryllodes sigillatus*), the life stage at harvest (such as adult or nymph), and the rearing diet (Zielińska et al. 2015; Kilburn et al. 2020). Besides being a rich source of indispensable AA (IAA), CKP contains up to 18% fat on a DM basis (Makkar et al. 2014; Valdés et al. 2022), along with a notable content of B-group vitamins (Schmidt et al. 2019). Chitin, which is the main component of arthropod exoskeletons, is also a significant component of CKP and is known for its potential gut health benefits (Lopez-Santamarina et al. 2020).

Due to its rich nutrient profile and the environmental benefits of insect farming compared to traditional livestock production (Li et al. 2023; Zafar et al. 2024), CKP has gained attention as a promising dietary ingredient for both humans (Stull et al. 2018; Ho et al. 2022) and various livestock species (Wang et al. 2005; Boontiam et al. 2022; Khonkhaeng et al. 2025). In healthy adult humans, daily intake of 25 g of CKP supported the growth of *Bifidobacterium animalis* in the gut and lowered plasma concentrations of tumor necrosis factor-alpha, indicating potential benefits for gut health and metabolism (Stull et al. 2018). Studies in poultry (Khan 2018) and swine (Boontiam et al. 2022) have reported that CKP can replace conventional protein sources, such as soybean meal and fish meal, without negatively impacting growth performance, feed efficiency, or nutrient digestibility.

These findings encourage further scientific investigation of CKP as a dietary ingredient for other species, such as domestic dogs. No detrimental effects were observed when including CKP (24% as-fed) in extruded dog food on fecal microbiome (Jarett et al. 2019) and on short-term health and nutrient digestibility (Areerat et al. 2021). However, while Kilburn et al. (2020) found that CKP inclusion up to 24% (as-fed) in extruded diets did not negatively impact short-term health or diet palatability, levels above 8% (as-fed) reduced apparent total tract digestibility (ATTD) of CP.

Although some studies have evaluated CKP as a dietary ingredient in dog food (Jarett et al. 2019; Kilburn et al. 2020; Areerat et al. 2021), there is limited data on the protein quality and metabolic effects of this ingredient, both of which are essential for formulating diets that support the long-term health of dogs. Protein quality is defined as the ability of a food source to meet the individual’s IAA requirement (Schaafsma 2005). Crucially, ATTD of CP does not provide information about the amount of IAA from an ingredient or a complete diet that is available for metabolic processes. One study assessed the protein quality of CKP for dogs using an *in vitro* digestion model and found that CKP had a protein digestibility-corrected AA score comparable to that of fish meal and greater than that of poultry meal (PM) (Bosch et al. 2014).

The Food and Agriculture Organization recognizes the digestible IAA score (DIAAS) as the current gold standard for evaluating protein quality in human foods. This method is also used to evaluate protein quality in pet food ingredients, with the ileal-cannulated swine and cecectomized rooster models being the most commonly used animal models for such evaluations (Oba et al. 2023). Typically, ingredients with a DIAAS above 75 are regarded as good-quality protein sources (Herreman et al. 2020). In fact, Malla et al. (2022) assessed the protein quality of crickets for humans using the ileal-cannulated swine model and reported that crickets had a DIAAS greater than 100 for adult humans, classifying them as an “excellent quality” protein. However, it is important to note that DIAAS is a static measure and does not consider protein kinetics, which can significantly influence metabolism (Wolfe et al. 2021). Protein sources with similar CP and gross AA concentrations, as well as DIAAS values, may still differ in their kinetics, and consequently, in their metabolic effects (Pennings et al. 2011; Burd et al. 2012; Areta et al. 2013). Assessing postprandial plasma AA response can provide additional insight into the rate and levels at which AA appear in the plasma pool after a meal, which has an impact on key physiological processes, including protein synthesis, carbohydrate metabolism, and hormone regulation (van Dam et al. 2024; Yimam et al. 2025). Studies in humans have demonstrated that different protein sources can elicit distinct postprandial AA responses, leading to variable effects on muscle protein synthesis (Pennings et al. 2011; Burd et al. 2012; Trommelen et al. 2019).

Despite being a promising ingredient in dog diets, there is a lack of data on the effects of CKP on postprandial AA and carbohydrate responses. Additionally, as some AA can modulate insulin secretion and subsequent glucose clearance, assessing these parameters simultaneously provides a more comprehensive view of how a novel protein impacts metabolism. We hypothesized that dogs fed a CKP-based diet have similar postprandial AA, glucose, and insulin responses compared to those fed a PM-based diet. Therefore, this study aimed to examine the effects of replacing PM (30% inclusion, as-fed basis) with CKP in extruded dog diets on postprandial plasma AA, glucose, and insulin responses in healthy adult dogs.

## Materials and methods

### Animals and housing

The study was conducted at Kansas State University (Manhattan, KS) over a 28-d period during July and August 2024. All procedures were reviewed and approved by the Institutional Animal Care and Use Committee at Kansas State University under protocol #4968. Eight castrated beagles (4 males and 4 females) of similar age (3.89 ± 0.02 yr, mean ± SD) and initial body weight (11.0 ± 2.28 kg, mean ± SD) were pair-housed in kennels (2.6 × 3 m) at the Large Animal Research Center facility in Manhattan, KS. The housing environment was temperature-controlled (22 to 23 °C) with a 16:8 h light:dark cycle. When weather permitted, the dogs had outdoor access from 0900 to 1500 h daily in a fenced, concrete-surface area equipped with enrichment items, such as a sand pond, canopy, and slides.

### Ingredient and experimental diets

CKP was supplied by Entomo Farms (Norwood, Ontario, Canada) and was produced from roasted banded crickets (*Gryllodes sigillatus*) at the adult stage (5 to 6 wk old), whereas PM was sourced from Fairview Mills (Seneca, KS). The CKP and PM ingredients were analyzed for moisture (AOAC 930.15), ash (AOAC 942.05), crude fat by acid hydrolysis (AOAC 954.02), crude fiber (AOCS Ba 6a-05), AA concentrations (AOAC 994.12, with performic acid oxidation prior to the analysis of methionine and cystine; and AOAC 988.15 for tryptophan), and CP as nitrogen using 6.25 as the nitrogen to protein conversion factor (AOAC 990.03) at a commercial analytical laboratory (Midwest Laboratories, Omaha, NE). For CKP, the CP was also estimated using 5.25 conversion factor to account for non-protein nitrogen (Boulos et al. 2020). The proximate composition and AA contents of the aforementioned ingredients are presented in Table 1.

**Table 1:** Analyzed macronutrient and amino acid concentrations of the poultry meal (PM) and cricket powder (CKP) ingredients utilized to produce experimental diets.

Nutrient	PM	CKP
**Dry matter, % **	93.2	95.0
**% dry matter basis**		
**Crude protein^1^**	68.7	71.2
**Crude fat**	13.8	18.1
**Crude fiber**	2.55	9.58
**Ash**	9.79	7.36
**Indispensable amino acids**		
**Arginine**	4.56	4.47
**Histidine**	1.50	1.47
**Isoleucine**	2.28	2.12
**Leucine**	5.28	4.74
**Lysine**	4.38	3.46
**Methionine**	1.25	0.97
**Phenylalanine**	2.54	2.17
**Threonine**	2.53	2.38
**Tryptophan**	0.67	0.68
**Valine**	2.55	2.99
**Dispensable amino acids**		
**Alanine**	4.28	5.72
**Aspartic acid**	5.53	6.19
**Cystine**	0.61	0.55
**Glutamic acid**	8.36	7.41
**Glycine**	6.11	3.44
**Proline**	3.70	3.73
**Serine**	2.69	3.04
**Tyrosine**	2.15	3.13
**Sum of amino acids**	60.97	58.66

1 Calculated using 6.25 as the conversion factor from nitrogen to crude protein. If using a conversion factor of 5.25 for cricket powder, a crude protein content of 59.8% is estimated.

Two experimental diets were formulated to meet or exceed the Association of American Feed Control Officials Official Publication nutrient recommendations for adult dogs at maintenance (AAFCO 2023). The control diet (CTRL) used PM as the primary protein source at 30% inclusion on an as-fed basis, which was entirely replaced by CKP in the CKP diet (CKP-D) (Table 2). The inclusion levels of corn, corn protein meal, and chicken fat were adjusted in the CKP-D to ensure comparable metabolizable energy and CP contents between the two diets (Table 3). PM and CKP contributed 63.6% and 62.4%, respectively, of the total CP in the CTRL and CKP-D diets. Both diets were produced using a 37.3 kW (50 hp) pilot-scale single screw extruder (X-20, Wenger Manufacturing, Sabetha, KS) and dried by a pilot-scale gas-fired continuous dryer (Series 4800, Wenger Manufacturing, Sabetha, KS) until moisture content was less than 10%. Detailed information on raw material sourcing and diet production is provided by Chen et al. (2025).

**Table 2:** Ingredient inclusion of the control (CTRL) and cricket powder (CKP-D) experimental diets.

Ingredient, % as-fed	CTRL	CKP-D
**Corn**	42.4	49.6
**Beet pulp**	3.80	3.80
**Poultry meal**	30.0	–
**Cricket meal**	–	30.0
**Corn protein meal, 60% CP^1^**	15.8	12.5
**Chicken fat**	6.0	2.0
**Dry dog flavoring**	1.0	1.0
**Calcium carbonate**	0.15	0.15
**Potassium chloride**	0.15	0.15
**Salt**	0.25	0.25
**Vitamin E, 50% dry**	0.1	0.1
**Choline chloride, 60% dry**	0.1	0.1
**Trace mineral premix^2^**	0.1	0.1
**Vitamin premix^3^**	0.15	0.15
**Total**	100	100

1 Crude protein.

2 Trace mineral premix: 0.66% moisture, 21.5% calcium, 0.02% sodium, 0.57% magnesium, 38,910 mg/kg iron, 11,234 mg/kg copper, 5842 mg/kg manganese, 88,000 mg/kg zinc, 1584 mg/kg iodine, 310 mg/kg selenium, 19% nitrogen-free extract, and 1% crude fat.

3 Vitamin premix: 5.51% moisture, 4.02% crude protein, 34.5% ash, 13.4% calcium, 17,162,999 IU/kg vitamin A, 920,000 IU/kg vitamin D, 79,887 IU/kg vitamin E, 14,252 mg/kg thiamine, 4719 mg/kg riboflavin, 12,186 mg/kg pantothenic acid, 64,736 mg/kg niacin, 5537 mg/kg pyridoxine, 720 mg/kg folic acid, 70 mg/kg biotin, 22 mg/kg vitamin B12.

**Table 3:** Analyzed nutrient composition and calculated metabolizable energy content of the control (CTRL) and cricket powder (CKP-D) experimental diets.

Nutritional composition	CTRL	CKP-D
**Dry matter, % **	92.2	92.9
**% dry matter basis**		
**Crude protein^1^**	33.4	35.3
**Crude fat**	12.2	9.8
**Ash**	5.0	3.5
**Total dietary fiber**	10.0	11.0
**Soluble fiber**	2.7	3.1
**Insoluble fiber**	7.3	7.9
**Crude fiber**	1.5	4.2
**Nitrogen-free extract^2^**	40.3	43.4
**Total starch**	37.9	39.0
**Energy**		
**Metabolizable energy,^3^ kcal/g**	3.61	3.59
**Indispensable amino acids**		
**Arginine**	1.77	1.84
**Histidine**	0.78	0.86
**Isoleucine**	1.32	1.39
**Leucine**	3.38	3.57
**Lysine**	1.69	1.61
**Methionine**	0.72	0.64
**Methionine + Cystine**	1.10	1.01
**Phenylalanine**	1.67	1.66
**Phenylalanine + Tyrosine**	2.95	3.38
**Threonine**	1.04	1.02
**Tryptophan**	0.26	0.26
**Valine**	1.61	1.81
**Dispensable amino acids**		
**Alanine**	2.34	2.84
**Aspartic acid**	2.71	2.97
**Cystine**	0.38	0.37
**Glutamic acid**	5.03	4.97
**Glycine**	2.26	1.64
**Proline**	2.31	2.40
**Serine**	1.50	1.70
**Tyrosine**	1.28	1.72
**Sum of total amino acids**	32.05	33.27

1 Calculated using 6.25 as the conversion factor from nitrogen to crude protein. Using a factor of 5.25 for cricket powder, the crude protein content totals 31.90%.

2 Nitrogen-free extract is calculated as 100 − (crude protein% + crude fat% + total dietary fiber% + ash% + moisture%).

3 Calculated using the modified Atwater factors, metabolizable energy (kcal/kg) = 10 × ([3.5 × crude protein%, as fed] + [8.5 × crude fat%, as fed] + [3.5 × nitrogen-free extract%, as fed]).

Experimental diets were analyzed for moisture (AOAC 930.15), ash (AOAC 942.05), CP as nitrogen using 6.25 as the nitrogen to protein conversion factor (AOAC 990.03), crude fat by acid hydrolysis (AOAC 954.02), crude fiber (AOCS Ba 6a-05), and total dietary fiber (AOAC 991.43) and AA concentrations using the same procedures described previously at the same commercial laboratory used for ingredient analysis. Total starch was analyzed using the Total Starch Assay Kit (Megazyme Inc., Wicklow, Ireland) following the manufacturer’s instructions to determine the total starch content of samples containing resistant starch. Metabolizable energy (ME, kcal/kg as fed) of experimental diets was estimated using modified Atwater factors as follows:


ME (kcal/kg)=10×([3.5×CP, % as fed]       +[8.5×crude fat, % as fed]       +[3.5×nitrogen-free extract, % as fed])

where nitrogen-free extract % (as fed) was calculated as 100 − (CP% + crude fat% + total dietary fiber% + ash% + moisture%).

### Study design and blood collection

Pair-housed dogs were randomly assigned to two groups, and the study was conducted as a crossover design. The study consisted of a 7-d wash-in period that preceded the experimental phase, which consisted of two 7-d experimental periods separated by a 7-d wash-out period in between them. During the wash-in and wash-out periods, dogs were fed CTRL. The two dogs that were pair-housed in the same kennel received the same diet during each period.

Dogs had *ad libitum* access to water, and food was provided twice daily in equal rations at 0800 and 1600 h with pair-housed dogs separated during feeding. At the beginning of the study, dogs were fed based on individual records of ME intake. Body weight was monitored weekly in the morning before the start of each period, and food adjustments were made as needed to maintain body weight throughout the study.

On the last day of each experimental period, a 12-h postprandial meal response test was conducted. On the morning of blood collection, a forearm on each dog was shaved to expose the cephalic vein and treated with topical anesthetic cream (Alembic Pharmaceuticals Inc., Bedminster, NJ) before catheter placement. After approximately 30 min, the area was cleaned, and a 20-gauge intravenous catheter (Exel Safelet Catheter ref#26741; Exelint International, Redondo Beach, CA) was placed and connected to a one-way stopcock (World Precision Instruments, Sarasota, FL). The catheter was then locked with 0.3 mL heparinized saline (50 IU/mL; Sagent Pharmaceuticals, Schaumburg, IL), and a fasted (∼16 h) blood sample (3 mL) was collected. Dogs were then fed their morning ration (50% of their total daily food allowance), and 15 minutes were allowed for food consumption after they started eating. Subsequent blood samples (∼3 mL) were collected into 5-mL sodium heparin-coated tubes (Becton, Dickinson and Company, Franklin Lakes, NJ) at 5, 10, 15, 30, 60, 90, 120, 180, 240, 300, 360, 420, 480, 540, 600, 660, and 720 min after the start of the meal. At each time point, the initial 0.3 mL of blood was withdrawn and discarded to clear the catheter dead space. After each sampling, the catheter was re-locked with 0.3 mL heparinized saline (50 IU/mL). Samples were briefly stored on ice (<1 h) until centrifugation at 1,500 × *g* for 15 min at 4 °C (AccuSpin Micro 17R, Thermo Fisher Scientific, Waltham, MA). Plasma was immediately stored at −20 °C on site and, at the end of the sampling day, transported on dry ice to be stored at -80 °C until analysis.

### Glucose, insulin, and AA analyses

Plasma glucose concentrations were measured using the glucose colorimetric assay kit (#10009582; Cayman Chemicals, Ann Arbor, MI) according to the manufacturer’s instructions. Plasma insulin concentrations were analyzed using the canine insulin ELISA kit (#10-1203-01; Mercodia, Uppsala, Sweden) according to the manufacturer’s instructions. The intra-assay coefficients of variation for glucose and insulin were 4.0% and 5.24%, respectively. Results for glucose and insulin in fasted blood (Time 0) samples were used to calculate the homeostatic model assessment of insulin resistance (HOMA-IR) as described by Respondek et al. (2008) with the calculation HOMA-IR = (insulin (mU/mL) × glucose (mM)) / 22.5.

For AA analysis, 100 µL of 10% sulfosalicylic acid (w/v, containing the internal standard norvaline) was added to an equal volume of plasma samples for deproteinization. The samples were incubated on ice for 1 h and centrifuged at 17,000 × *g* for 25 min at 4 °C. AA standards and deproteinized samples were derivatized using the AccQ-Tag Ultra Derivatization Kit (Waters Corporation, Milford, MA). The derivatized samples were then analyzed using ultra-performance liquid chromatography (Acquity UPLC H-Class PLUS System; Waters Corporation, Milford, MA). Briefly, 1 µL of sample was injected and separated by Accq Tag Ultra C18 Column (2.1 × 100 mm, 1.7 µm; Waters Corporation) that was maintained at 43 °C, and AA were detected by UV absorbance (260 nm). The sample peaks and peaks from a known standard were interpreted by Empower 3 software (Waters Corporation). IAA was calculated as the sum of Arg, His, Ile, Leu, Lys, Met, Phe, Thr, Trp, and Val. Dispensable AA (DAA) was calculated as the sum of Ala, Asn, Gln, Glu, Gly, Orn, Pro, Ser, Tau, and Tyr. Branched chain AA was calculated as the sum of Leu, Ile, and Val. Total AA was calculated as the sum of IAA and DAA.

Incremental area under the curve (iAUC = total positive area above baseline) and net area under the curve (NetAUC = iAUC − total negative area below baseline) were calculated for the glucose, insulin, and AA data using GraphPad Prism (version 10.2.0; GraphPad Software, San Diego, CA). Maximum concentration (Cmax) was defined as the highest observed plasma concentration following feeding, and the time to reach Cmax (Tmax) was the time point at which Cmax occurred.

### Statistical analysis

Different models were used according to the groups of dependent variables analyzed. The AUC-derived responses were analyzed using the following linear mixed model:


$$Y_{\mathrm{iklm}}=\mu +T_{i}+G_{k}+P_{l}+d_{m}+e_{\mathrm{iklm}}$$

where $Y_{\mathrm{iklm}}$ is the observed value; μ is the intercept; $T_{i}$ is the fixed effect of the *i*^th^ level of treatment, with *i *= 1,2; $G_{k}$ is the fixed effect of the *k*^th^ level of group, with *k *= 1,2; $P_{l}$ is the fixed effect of the *l*^th^ level of period, with *l *= 1,2; $d_{m}$ is the random effect of the *m*^th^ dog, with *m *= 1…7, assuming $d∼N(0,I_{d}\sigma_{d}^{2})$, where $I_{d}$ represents the identity matrix of dimension equal to the number of dogs, and $\sigma_{d}^{2}$ is the dog variance; and $e_{\mathrm{iklm}}$ is the random error associated with $y_{\mathrm{iklm}}$, assuming $e∼N(0,I_{e}\sigma_{e}^{2})\;$where $I_{e}$ represents the identity matrix of dimension equal to the number of observations, and $\sigma_{e}^{2}$ is the residual variance. For glucose, insulin, and AA concentrations, dogs had multiple samples collected within a period. Hence, the model described above was further expanded, as:


$$Y_{\mathrm{ijklm}}=\mu +T_{i}+M_{j}+{\left(TM\right)}_{ij}+G_{k}+P_{l}+d_{m}+{\left(Td\right)}_{im}+e_{\mathrm{ijklm}}$$

where $Y_{\mathrm{ijklm}}$ is the observed value; μ, $T_{i}$, $G_{k}$, $P_{l}$, and $d_{m}$ are as previously defined; $M_{j}$ is the fixed effect of the *j*^th^ level of measurement, with *j *= 1…18; ${\left(TM\right)}_{ij}$ is the fixed effect interaction between $T_{i}$ and $M_{j}$; ${\left(Td\right)}_{im}$ is the random effect interaction between $T_{i}$ and $d_{m}$, assuming $Td∼N(0,I_{\mathrm{Td}}\sigma_{Td}^{2})$, where $I_{\mathrm{Td}}$ represents the identity matrix of dimension equal to the number of dogs times the number of treatments, and $\sigma_{Td}^{2}$ is the treatment-dog variance; and $e_{\mathrm{ijklm}}$ is the random error associated with $y_{\mathrm{ijklm}}$, assuming $e∼N(0,{\mathrm{AR}(1)\sigma }_{e}^{2})\;$where AR(1) represents the first-order autoregressive structure, and $\sigma_{e}^{2}$ is the residual variance. Different variance-covariance structures were tested, but AR(1) was used for final analysis because it showed the lowest AIC values across most analyses. Prior to final analysis, residuals were evaluated for normality and homoscedasticity. For each analysis, data points were removed one at a time until absolute Studentized residuals were lower than 3 and had non-significant (*P *> 0.05) Shapiro-Wilk’s test for normality. Expected means were generated from the final models and separated when significant (*P *< 0.05). A slice option was utilized to separate the effects of treatment and time when an interaction was considered significant. All analyses were performed in SAS Studio 3.81 (Enterprise Edition, SAS Institute Inc., Cary, NC, USA).

## Results

One dog was removed from the study at the end of the wash-in period for reasons unrelated to the experimental diets. Dogs maintained body weight throughout the study, with no differences (*P *> 0.05) observed between dietary treatments (CTRL: 11.00 kg vs. CKP-D: 10.98 kg). Similarly, daily food intake (CTRL: 227 g/d vs. CKP-D: 230 g/d) did not differ (*P *> 0.05) between treatment groups. On sample collection days, all dogs consumed their morning ration within 15 min of the timer starting, except for one dog on the CKP-D diet during experimental period 1, which finished the meal within 30 min. This dog was still considered for the study due to a small sample size.

### Plasma glucose and insulin

**Figure 1:**
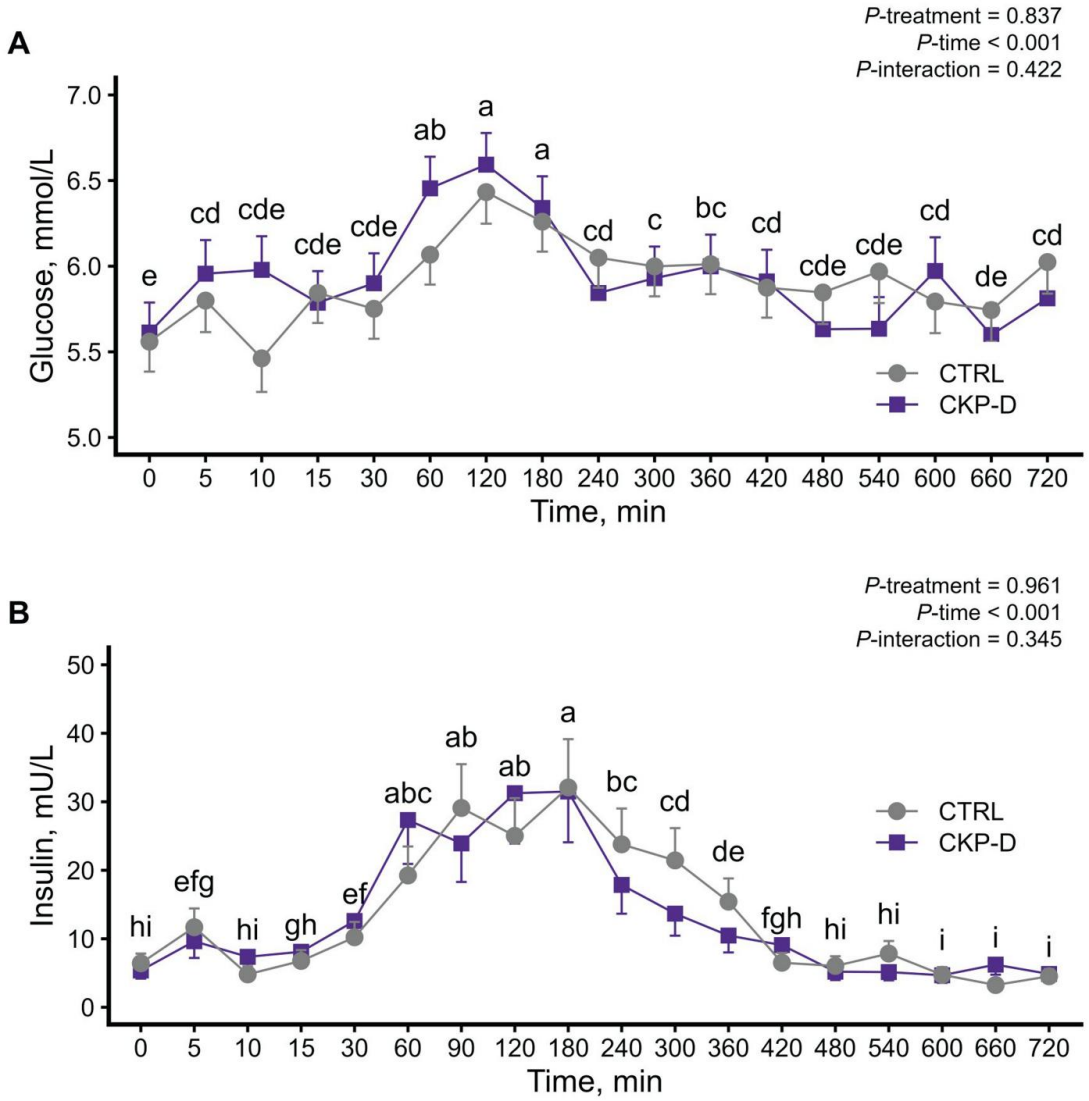
Mean plasma (**A**) glucose and (**B**) insulin concentrations from fasted (0 min) to 720 min after a meal in healthy dogs fed either control (CTRL) or cricket powder diet (CKP-D). Values are presented as least squares means ± SEM. Time points that do not share a common letter are significantly different from each other (*P*-time < 0.05).

As presented in Figure 1, the responses of glycemia and insulin were not influenced by the dietary treatment (*P *> 0.05), and no treatment × time (*P *> 0.05) interaction was observed. However, both glucose and insulin levels changed over time following a meal (*P *< 0.05). LSMeans and SEM are provided in Table S1. Plasma glucose concentrations remained similar from the fasted state through 30 min post meal, except at 5 min post meal, which was greater than fasted (*P *< 0.05). Glucose concentrations increased thereafter, peaking between 60 and 180 min (*P *< 0.05). After the peak, glucose concentrations declined toward fasted levels by 480 min post meal and was again greater than fasted at 600 and 720 min post meal (*P *< 0.05). Similar to glucose, plasma insulin concentrations remained similar from the fasted state through 15 min post meal, except at 5 min post meal, which was greater than fasted (*P *< 0.05). Insulin concentrations increased thereafter, peaking between 60 and 180 min post meal (*P *< 0.05). After the peak, insulin concentrations declined, achieving concentrations similar (*P *> 0.05) to fasted levels by 420 min post meal.

No differences (*P *> 0.05) were observed for the HOMA-IR, iAUC, NetAUC, or Tmax for plasma glucose (Table 4). However, the glucose Cmax was 4.7% greater in dogs fed CKP-D compared to those fed CTRL (*P *< 0.05; Table 4). For plasma insulin, no differences were observed (*P *> 0.05) between treatment groups for iAUC, NetAUC, Tmax, or Cmax.

**Table 4:** Area under the curve parameters^1^ for plasma glucose and insulin in healthy dogs consuming the control (CTRL) or cricket powder (CKP-D) experimental diets.

Trait	Parameter^2^	Treatment		*P*-value^4^
		CTRL	CKP-D	
**HOMA-IR^3^**		1.31 (0.23)	1.61 (0.29)	0.419
**Glucose**	iAUC, mM·min	279 (72)	317 (77)	0.698
	NetAUC, mM ·min	244 (99)	233 (105)	0.935
	Cmax, mM	6.63 (0.10)	6.94 (0.11)	**0.017**
	Tmax, min	226 (73)	230 (78)	0.969
**Insulin**	iAUC, (mU/L)·min	8535 (1624)	6816 (1736)	0.488
	NetAUC, (mU/L)·min	8279 (1696)	6667 (1814)	0.543
	Cmax, mU/L	58.6 (10.6)	44.5 (11.3)	0.389
	Tmax, min	182 (31)	121 (32)	0.067

1 Least square mean (SEM).

2 iAUC, incremental area under the curve, represents the total positive area above baseline; NetAUC, net area under the curve, is calculated as iAUC minus the total negative area below baseline; Cmax, maximum concentration, is the highest observed plasma concentration following feeding; Tmax, time to Cmax, is the time point at which Cmax occurred.

3 Homeostatic model assessment for insulin resistance.

4 Bolded *P*-values indicate that means within the same row differ significantly with respect to the diet effect at *P* < 0.05.

### Plasma AAs

Plasma concentrations of IAA are presented in Table 5. Main effects of treatment (*P *< 0.05) and time (*P *< 0.05) were observed for all IAA, but a treatment × time interaction was detected (*P *< 0.05) only for Phe and Trp. Plasma concentrations of all IAA were at least 16% greater in dogs (calculated as a mean for comparison in all timepoints) fed the CKP-D compared to those fed the CTRL, with Ile, Leu, Phe, Val being more than 25% greater for CKP-D than for CTRL (*P *< 0.05). This greater AA concentration in dogs fed CKP-D was also reflected in summed pools of total AA, total IAA, and total branched-chain AA (BCAA), which were 16%, 25%, and 31% greater, respectively, in dogs fed CKP-D than those fed CTRL (*P *< 0.05). Dogs fed CKP-D had 9% greater DAA than those fed CTRL (*P *< 0.05).

**Table 5:** Postprandial response of plasma indispensable amino acids (µM)^1^ in dogs consuming the control (CTRL) or cricket powder (CKP-D) experimental diets.

AA^2^	Treatment	Time, min									SEM^3^	*P*-value		
		0	30	60	90	120	180	240	300	360		Trt	Time	Trt×Time
**Arg**	CTRL	194^c,d^	193^d^	208^b,c,d^	210^a,b,c^	202^a,b,c^	216^a^	221^a,b^	226^a^	201^a,b,c,d^	11	**<0.001**	**0.012**	0.645
	CKP-D	224^c,d^	214^d^	226^b,c,d^	240^a,b,c^	249^a,b,c^	267^a^	244^a,b^	254^a^	244^a,b,c,d^	12			
**His**	CTRL	112^e,f^	101^f^	111^e^	117^d^	123^c^	134^a^	136^a,b^	139^a,b^	131^b,c^	5	**<0.001**	**<0.001**	0.136
	CKP-D	133^e,f^	134^f^	139^e^	149^d^	162^c^	173^a^	164^a,b^	160^a,b^	160^b,c^	5			
**Ile**	CTRL	75^f^	77^f^	98^e^	108^c,d^	115^a,b^	123^a^	120^b,c^	112^c,d^	101^de^	9	**<0.001**	**<0.001**	0.303
	CKP-D	108^f^	108^f^	126^e^	150^c,d^	169^a,b^	175^a^	152^b,c^	143^c,d^	137^d,e^	9			
**Leu**	CTRL	124^f^	129^f^	197^e^	245^c,d^	269^a,b^	292^a^	282^b,c^	259^d^	225^d^	21	**<0.001**	**<0.001**	0.358
	CKP-D	173^f^	188^f^	235^e^	313^c,d^	372^a,b^	383^a^	330^b,c^	300^d^	273^d^	22			
**Lys**	CTRL	171^b,c,d^	170^c,d^	191^b,c,d^	189^a,b^	184^a,b^	188^a^	188^a,b,c^	184^a,b,c^	158^d^	11	**0.005**	**0.023**	0.384
	CKP-D	220^b,c,d^	210^c,d^	212^b,c,d^	226^a,b^	231^a,b^	250^a^	221^a,b,c^	221^a,b,c^	214^d^	12			
**Met**	CTRL	71^d^	67^d^	75^d^	83^c^	90^b^	103^a^	112^a^	116^a^	108^a^	6	**0.003**	**<0.001**	0.177
	CKP-D	87^d^	82^d^	82^d^	101^c^	111^b^	125^a^	118^a^	117^a^	115^a^	7			
**Phe**	CTRL	66^B,c^	58^B,d^	69^B,b,c^	73^B,a,b^	76^B,a^	78^B,a^	78^B,a^	77^B,a^	69^B,bc^	3	<0.001	<0.001	**0.038**
	CKP-D	83^A,d,e^	82^A,d,e^	87^A,c,d,e^	93^A,b^	102^A,a^	105^A,a^	91^A,b,c^	94^A,b,c^	86^A,d,e^	3			
**Thr**	CTRL	262^d,e,f^	247^f^	268^e,f^	270^d,e^	269^c,d^	286^b,c^	302^a,b^	319^a^	304^a,b^	31	**0.001**	**<0.001**	0.535
	CKP-D	321^d,e,f^	298^f^	307^e,f^	332^d,e^	353^c,d^	368^b,c^	370^a,b^	380^a^	384^a,b^	32			
**Trp**	CTRL	90^B,f^	93^B,f^	109^B,e^	110^B,e^	111^B,c,d^	118^B,a,b,c,d^	122^a,b^	122^a,b,c^	117^a,b,c,d^	6	0.007	<0.001	**0.046**
	CKP-D	114^A,f^	128^A,d,e^	137^A,b,c,d^	144^A,a,b,c^	148^A,a,b^	149^A,a^	135^c,d,e^	133^c,d,e^	130^d,e^	6			
**Val**	CTRL	170^f^	167^f^	205^e^	228^c,d^	244^b^	266^a^	263^b^	250^b,c^	223^d^	17	**<0.001**	**<0.001**	0.175
	CKP-D	224^f^	223^f^	256^e^	308^c,d^	354^b^	366^a^	334^b^	312^b,c^	289^d^	18			
**Total AA**	CTRL	3466^c,d^	3447^d^	3875^c^	4069^b^	4112^b^	4376^a^	4428^a,b^	4497^a,b^	4131^b^	185	**<0.001**	**<0.001**	0.505
	CKP-D	4152^c,d^	3949^d^	4219^c^	4774^b^	5039^b^	5272^a^	4933^a,b^	4911^a,b^	4776^b^	197			
**Total BCAA**	CTRL	368^f^	372^f^	500^e^	581^c,d^	628^a,b^	681^a^	665^b^	621^c^	549^d^	47	**<0.001**	**<0.001**	0.281
	CKP-D	506^f^	519^f^	617^e^	772^c,d^	896^a,b^	924^a^	817^b^	755^c^	699^d^	49			
**Total IAA**	CTRL	1335^e^	1330^e^	1532^d^	1634^b,c^	1683^a,b,c^	1803^a^	1825^a,b^	1805^a,b,c^	1637^c^	101	**<0.001**	**<0.001**	0.336
	CKP-D	1701^e^	1677^e^	1815^d^	2112^b,c^	2255^a,b,c^	2373^a^	2166^a,b^	2113^a,b,c^	2044^c^	105			

a–f Means within the same row lacking the same letter differ significantly with respect to the time effect at *P *< 0.05.

A,B Means within the same column lacking the same letter differ significantly between diets with respect to the interaction effect at *P *< 0.05.

1 Least square means.

2 AA, amino acid; BCAA, branched chain amino acids; IAA, indispensable amino acids.

3 Pooled SEM. Individual SEM for each variable is presented as supplementary material (Table S2).

Relative to baseline (time 0), ingestion of the diets was followed by a change in plasma total IAA during the postprandial period. All IAA increased from 0 to 180 min postprandially (*P *< 0.05). From 180 to 360 min, Arg and Met remained relatively stable (*P *> 0.05), whereas Ile, Leu, Val, and His decreased (*P *< 0.05) but stayed above baseline at 360 min. Arg and Lys concentration returned to baseline (*P *> 0.05) at 360 min. For Trp, CKP-D showed greater (*P *< 0.05) concentrations than CTRL until 180 min, but both treatments were similar (*P *> 0.05) thereafter. Moreover, Trp increased (*P *< 0.05) in the CTRL from 0 to 180 min and remained stable (*P *> 0.05) after that. However, for the CKP-D, Trp increased (*P *< 0.05) from 0 to 180 min but, after this time, decreased (*P *< 0.05) until 360 min, without returning to baseline values. Dogs fed CKP-D had greater (*P *< 0.05) Phe concentrations than CTRL at all time points, but postprandial responses differed (*P *< 0.05) between treatments. For CTRL, Phe concentrations increased from 0 to 120 min, when Phe reached its peak, and remained elevated (*P *> 0.05) until 300 min, returning to baseline values at 360 min (*P *> 0.05). For dogs fed CKP-D, Phe increased (*P *< 0.05) from 0 to 120 min, then decreased (*P *< 0.05) at 240 min, returning to baseline at 360 min (*P *> 0.05). The summed IAA and BCAA concentrations followed similar responses to most AA, where it increased (*P *< 0.05) from 0 to 180 min and then decreased (*P *< 0.05) from 180 to 360 min but remained higher than baseline at 360 min (*P *< 0.05). Overall, most IAA peaked at 90, 120, or 180 min after consuming a meal, except Thr, which peaked at 240 min.

Plasma concentrations of DAA are presented in Table 6. The main effect of treatment was observed for all DAA (*P *< 0.05), except for Gly and Orn, and a time effect was observed for all DAA (*P *< 0.05), except Gln. Moreover, a treatment × time interaction was observed (*P *< 0.05) only for Glu and Gly. Plasma concentrations of DAA were significantly greater (*P *< 0.05) in dogs fed the CKP-D for Ala, Asn, Glu, Pro, and Ser, with mean increases of at least 15%, and Glu exhibited the greatest increase (50%). In contrast, concentrations of Gln and Tau were 12% and 13% greater (P < 0.05), respectively, in the CTRL group.

**Table 6:** Postprandial response of plasma dispensable amino acids (µM)^1^ in dogs consuming the control (CTRL) or cricket powder (CKP-D) experimental diets.

**AA** ^2^	Treatment	Time, min									**SEM** ^3^	*P*-value		
		0	30	60	90	120	180	240	300	360		Trt	Time	Trt×Time
**Ala**	CTRL	380^d,e^	378^e^	458^c,d^	481^a,b^	476^a^	458^a,b^	453^b,c^	468^a,b^	440^b,c^	24	**0.005**	**<0.001**	0.12
	CKP-D	534^d,e^	498^e^	499^c,d^	583^a,b^	601^a^	578^a,b^	553^b,c^	580^a,b^	553^b,c^	26			
**Asn**	CTRL	51^e^	55^d,e^	69^d^	75^c^	78^b,c^	89^a^	94^a^	99^a^	87^a,b^	5	**<0.001**	**<0.001**	0.252
	CKP-D	66^e^	69^d,e^	71^d^	97^c^	107^b,c^	115^a^	113^a^	110^a^	108^a,b^	6			
**Gln**	CTRL	795	774	745	740	723	764	754	766	735	45	**0.018**	0.364	0.385
	CKP-D	681	621	649	695	700	703	689	684	662	46			
**Glu**	CTRL	149^B,a^	137^B,b,c^	132^B,b,c^	135^B,b,c^	133^B,b,c^	140^B,a,b^	136^B,b,c^	132^B,b,c^	130^B,c^	6	<0.001	<0.001	**<0.01**
	CKP-D	232^A,a^	187^A,f^	202^A,c,d,e^	196^A,f,e^	204^A,c,d^	218^A,b^	213^A,b,c^	204^A,c,d,e^	199^A,d,e^	7			
**Gly**	CTRL	231^B,f^	242^e,f^	285^d^	286^c,d^	283^d^	303^b,c^	325^A,a,b^	352^A,a^	317^b,c^	14	0.14	<0.001	**0.013**
	CKP-D	277^A,a,b,c,d^	255^d,e^	257^d,e^	262^c,d,e^	273^b,c,d,e^	295^a,b,c^	290^B,a,b,c,d^	301^B,a,b^	310^a^	16			
**Orn**	CTRL	19^f^	22^e^	31^d^	37^a,b^	37^a^	39^a,b^	36^b,c^	35^c,d^	31^d^	2	0.627	**<0.001**	0.245
	CKP-D	20^f^	26^e^	29^d^	37^a,b^	41^a^	35^a,b^	34^b,c^	30^c,d^	28^d^	3			
**Pro**	CTRL	148^f^	170^f^	253^e^	289^d^	304^c,d^	346^a,b^	366^a,b^	385^a^	341^b,c^	21	**<0.001**	**<0.001**	0.242
	CKP-D	203^f^	220^f^	241^e^	335^d^	368^c,d^	398^a,b^	396^a,b^	401^a^	379^b,c^	22			
**Ser**	CTRL	161^d,e^	157^e^	173^d,e^	181^c,d^	182^c^	198^b^	217^a,b^	237^a^	223^a,b^	13	**<0.001**	**<0.001**	0.46
	CKP-D	234^d,e^	217^e^	225^d,e^	236^c,d^	254^c^	283^b^	272^a,b^	285^a^	293^a,b^	13			
**Tau**	CTRL	141^a,b^	117^e^	135^c,d^	144^a,b^	142^a,b^	156^a^	141^a,b,c^	140^b,c^	121^d,e^	8	**0.005**	**<0.001**	0.386
	CKP-D	137^a,b^	108^e^	114^c,d^	132^a,b^	137^a,b^	127^a^	121^a,b,c^	115^b,c^	110^d,e^	8			
**Tyr**	CTRL	54^d^	52^d^	60^d^	66^c^	70^a,b^	76^a^	76^a,b,c^	76^b,c^	68^c^	5	**<0.001**	**<0.001**	0.214
	CKP-D	68^d^	66^d^	71^d^	85^c^	102^a,b^	106^a^	92^a,b,c^	90^b,c^	86^c^	5			
**Total DAA**	CTRL	2,129^c^	2,115^c^	2,342^c^	2,434^b^	2,428^a,b^	2,571^a^	2,601^a,b^	2,691^a^	2,493^a,b^	98	**0.001**	**<0.001**	0.26
	CKP-D	2,452^c^	2,283^c^	2,299^c^	2,666^b^	2,789^a,b^	2,903^a^	2,770^a,b^	2,802^a^	2,737^a,b^	106			

a–f Means within the same row lacking the same letter differ significantly with respect to the time effect at *P *< 0.05.

A,B Means within the same column lacking the same letter differ significantly between diets with respect to the interaction effect at *P *< 0.05.

1 Least square means.

2 AA, amino acid; DAA, dispensable amino acids.

3 Pooled SEM. Individual SEM for each variable are presented as supplementary material (Table S3).

Sampling time affected all DAA in the plasma in the postprandial period (*P *< 0.05), except for Gln. From 0 to 90 min, concentrations of Ala and Orn increased, remained stable (*P *> 0.05) until 180 min, and decreased thereafter, but remained higher than baseline at 360 min. From 0 to 180 min, Asn and Pro concentrations increased, remaining stable from 180 to 300 min, with Asn still stable at 360 min, whereas Pro decreased at this time but remained above baseline values. Concentrations of Ser increased from 0 to 240 min and remained stable until 360 min. For Tau, concentration initially decreased from 0 to 30 min, then increased from 30 to 180 min, and then returned to the 30 min concentration by 360 min. Dogs fed the CTRL had greater (*P *< 0.05) Glu at all timepoints. Moreover, peak Glu concentrations were observed at baseline for both treatments. In dogs fed CTRL, Glu decreased (*P *< 0.05) at 30 min and then remained relatively stable through 360 min. In contrast, in dogs fed CKP-D, Glu also decreased (*P *< 0.05) at 30 min but subsequently increased (*P *< 0.05) from 30 to 360 min, without returning to baseline levels. For Gly, plasma concentrations were greater (*P *< 0.05) in dogs fed CKP-D at 0, 240, and 300 min, with the remaining timepoints having similar (*P *> 0.05) concentrations in both treatments. In dogs fed CTRL, Gly increased (*P *< 0.05) from 0 to 240 min, remained stable (*P *> 0.05) until 300 min, and then decreased (*P *< 0.05) at 360 min without returning to baseline. In contrast, Gly concentrations in dogs fed CKP-D remained relatively constant (*P *> 0.05) throughout the postprandial period, with no clear peak. The summed DAA concentrations increased (*P *< 0.05) from 0 to 120 min and then remained stable, with no significant decrease from 120 to 360 min. Overall, most DAA peaked at 90 or 180 min, except for Tyr, which peaked at 120 min and Ser, which peaked at 240 min.

**Figure 2:**
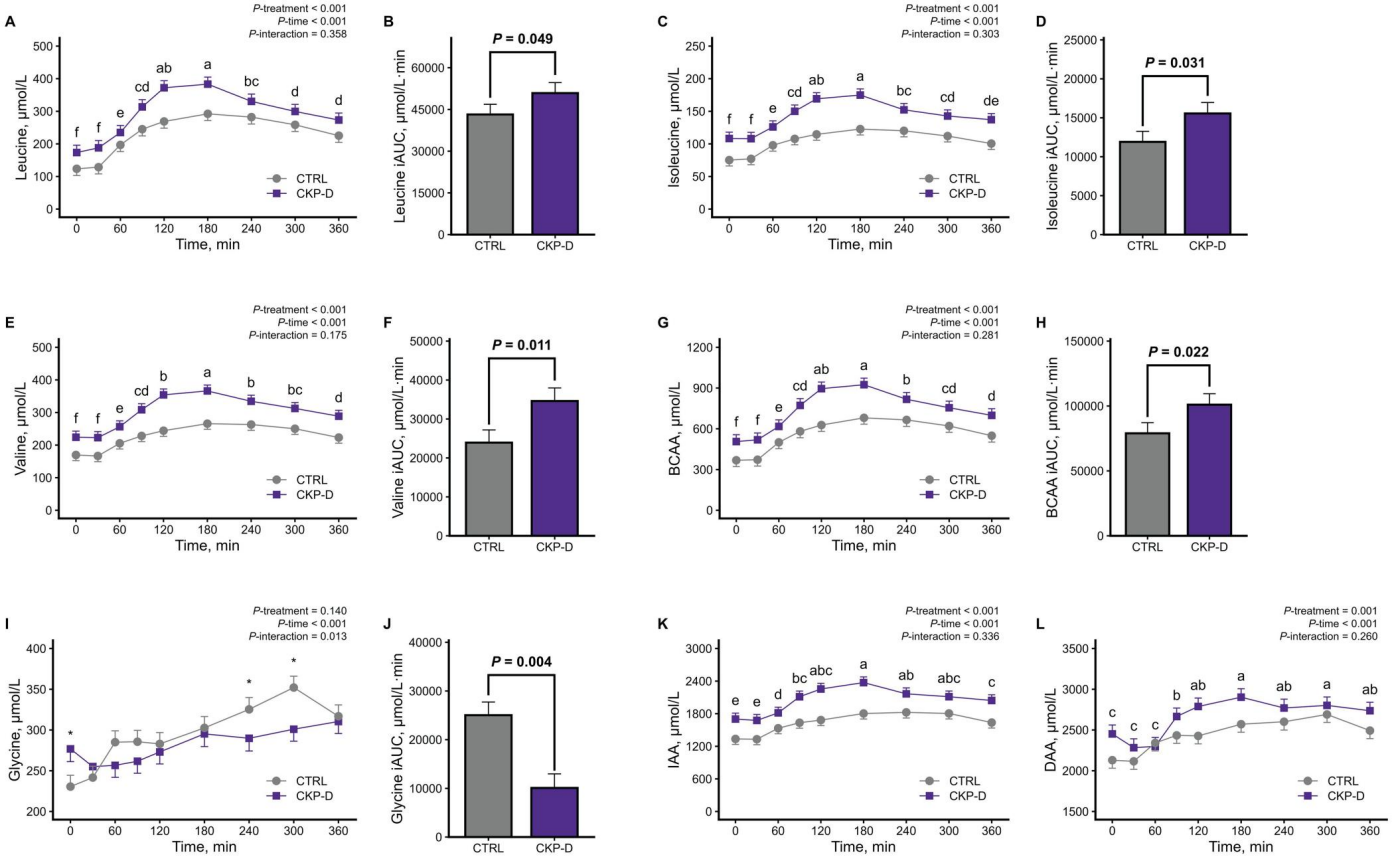
Mean plasma concentrations and incremental area under the curve (iAUC) of (**A, B**) leucine, (**C, D**) isoleucine, (**E, F**) valine, (**G, H**) total branched chain amino acids (BCAA) and (**I, J**) glycine in healthy dogs fed either control (CTRL) or cricket powder diet (CKP-D). The postprandial plasma concentrations of the sum of (**K**) total indispensable amino acids (IAA) and (**L**) total dispensable amino acids (DAA) are also presented. Values are presented as least squares means ± SEM. Time points that do not share a common letter in the postprandial response charts are significantly different from each other (*P*-time < 0.05). The asterisk indicates a difference between treatments at the specific time point (*P*-interaction < 0.05).

Although concentrations were greater for CKP-D for most AA, the AA iAUC was only different for 3 IAA, 1 DAA, and total BCAA (Table 7). Dogs fed CKP-D had greater (*P *< 0.05) iAUC values for Ile, Leu, Val, and summed BCAA (*P *< 0.05) compared to those fed CTRL (Figure 2). On the other hand, dogs fed the CTRL diet showed a greater (*P* < 0.05) iAUC for Gly than those fed the CKP-D diet.

**Table 7:** Effect of dietary treatment on incremental area under the curve (mM·min)^1^ for plasma amino acid concentrations over time in healthy dogs consuming the control (CTRL) or cricket powder (CKP-D) experimental diets.

Amino acid	Treatment		*P*-value^2^
	CTRL	CKP-D	
**Indispensable amino acids**			
** Arg**	6,862 (2,358)	12,118 (2,520)	0.186
** His**	5,313 (1,264)	7,999 (1,351)	0.180
** Ile**	11,908 (1,353)	15,555 (1,404)	**0.031**
** Leu**	43,187 (3,660)	50,874 (3,772)	**0.049**
** Lys**	5,921 (2,004)	6,296 (2,141)	0.898
** Met**	9,352 (1,578)	8,925 (1,685)	0.853
** Phe**	2,904 (860)	3,865 (919)	0.465
** Thr**	9,195 (3,103)	17,595 (3,317)	0.097
** Trp**	8,422 (1,693)	8,925 (1,810)	0.844
** Val**	23,928 (3,266)	34,611 (3,359)	**0.011**
**Dispensable amino acids**			
** Ala **	24,539 (4,161)	19,603 (4,449)	0.452
** Asn**	11,337 (1,040)	13,554 (1,095)	0.110
** Gln**	2,444 (7,607)	20,143 (8,130)	0.162
** Gly**	25,025 (2,702)	10,083 (2,889)	**0.004**
** Orn**	5,270 (511)	4,647 (518)	0.066
** Pro**	59,018 (2,626)	55,190 (2,716)	0.137
** Ser**	13,736 (2,428)	15,207 (2,594)	0.679
** Tau**	2,834 (954)	1,941 (1,020)	0.538
** Tyr**	5,415 (1,369)	8,967 (1,455)	0.091
**Sum of a group of amino acids**			
** Branched-chain amino acids**	78,974 (8,174)	101,042 (8,422)	**0.022**
** Indispensable amino acids**	122,574 (16,585)	157,368 (17,709)	0.186
** Dispensable amino acids**	122,768 (20,168)	125,805 (21,550)	0.919
**Total amino acids**	244,977 (33,539)	280,854 (35,829)	0.476

1 Least square mean (SEM).

2 Bolded *P*-values indicate that means within the same row differ significantly with respect to the diet effect at *P* < 0.05.

## Discussion

To the best of our knowledge, this is the first study to evaluate the postprandial AA, glucose and insulin responses in healthy beagles to an extruded diet formulated with CKP as the major protein source, compared with a diet formulated with PM at the same inclusion rate and balanced for CP content. Plasma glucose increased following meal consumption and was influenced only by postprandial time. When glucose concentrations peaked between 60 and 180 min after a meal, plasma insulin concentrations were also at their highest, as expected because insulin is the main regulator of glucose homeostasis and its secretion is closely associated with blood glucose concentrations (Norton et al. 2022). Similarly, Dai et al. (2022) evaluated the effects of feeding healthy humans either cricket or beef protein on postprandial blood glucose and insulin responses over a 300 min period. The authors reported no differences in glucose or insulin iAUC or glucose concentrations between treatments, but insulin concentrations at 30 min were greater in participants consuming the beef protein treatment (Dai et al. 2022). Although glucose and insulin dynamics over time were similar, the glucose Cmax was greater for CKP-D, likely because this diet contained more starch, which may have led to a greater release of glucose in the gut and a transiently greater peak concentration. Importantly, this modest increase in Cmax did not translate into a larger glucose iAUC, and the Tmax remained similar between diets. To further explore the relationship between glucose and insulin, HOMA-IR index was calculated. This index provides an indication of insulin production and tissue responsiveness, serving as an indicator of insulin resistance (Xenoulis et al. 2011). Consistent with the glucose and insulin results, HOMA-IR values were not affected by consumption of the different experimental diets.

Even though no major effects of diet on glucose and insulin were observed in the present study, other than the modestly greater Cmax for CKP-D than CTRL, insect-based food has been suggested to provide multiple health benefits due to the presence of bioactive compounds that may influence glucose metabolism. Escobar-Ortiz et al. (2022) reported that rats fed high-fat diets supplemented with 4% or 8% CKP from *Acheta domesticus* for 16 wk had improved fasting glucose and glucose tolerance, with reduced HOMA-IR values at the higher inclusion level. Moreover, Ahn et al. (2020) showed an antioxidative effect of extracted glycosaminoglycan from field cricket (*Gryllus bimaculatus*) in diabetic mice. Other studies have reported that chitin, a structural component found in insects, can lower glucose levels in the blood (Zheng et al. 2018) and that bioactive peptides derived from banded crickets may influence insulin secretion and glycemia (Acosta-Estrada et al. 2021). Similar effects were not observed in the current study, but this may be related to differences in the food matrix, the delivery form of CKP, or the amount of chitin present in the samples not being sufficient. For example, Zheng et al. (2018) provided chitin oligosaccharide in drinking water to mice consuming a high-fat diet, rather than including it as a part of the diet. Moreover, Ahn et al. (2020) administered field cricket glycosaminoglycan intraperitoneally in diabetic mice consuming a standard research mice diet. In contrast, in our study, concentration of chitin was not measured, and CKP was included as part of a complete extruded diet of where starch was a significant portion of the diet. Plasma glucose, insulin, and HOMA-IR values were within similar ranges of those reported for healthy dogs fed extruded diets in the fasted and postprandial periods (Holste et al. 1989; Banton et al. 2025; Hrițcu et al. 2025). Although some studies observed the potential of structural components of crickets in glucose regulation, a lack of effect of CKP in dog diets may also be attributed to the use of healthy dogs without impaired glucose metabolism. Further investigation of CKP in diets for dogs with glucose intolerance or diabetes is warranted.

Insulin release is also influenced by AA concentrations in the blood, but this effect is less than that of glucose (Banton et al. 2025). Some BCAA, such as leucine, stimulate insulin secretion (Yang et al. 2010). Thus, as iAUC for BCAA were greater for CKP-D than for CTRL, it was hypothesized that insulin, and consequently glucose, could be affected by diet; however, this was not observed in our study as discussed above. After its release, insulin’s role in the blood is not only to influence glucose uptake by tissues but also to enhance AA delivery to the muscles and stimulate protein anabolism (Fujita et al. 2006). Together with other hormonal signals and central nervous system regulation, insulin helps control the rates of protein synthesis, breakdown, and AA oxidation to maintain homeostasis (Loveday 2023), thereby affecting circulating AA concentrations. In the fasted state, AA in the blood primarily originates from tissue protein breakdown. However, after the consumption of the meal, the increase and subsequent decrease of plasma AA concentrations reflect the digestion, absorption, and metabolic clearance of dietary AA (Trommelen and van Loon 2024). Overall, all IAA increased in plasma following meal consumption for both experimental diets. Moreover, compared to CTRL, dogs fed the CKP-D had greater postprandial concentrations for all IAA, and iAUC was greater for BCAA (Leu, Ile, and Val). Although circulating hormone and nutrient concentrations measured over time provide a comprehensive understanding of postprandial metabolic responses following food ingestion, the iAUC quantifies the cumulative appearance of a nutrient in blood over a given period (Trommelen and van Loon 2024), indicating the plasma bioavailability of AA.

Because iAUC reflects the systemic availability of absorbed AA, differences in postprandial plasma responses may be influenced by the intrinsic digestibility and AA composition of dietary protein sources. Bosch et al. (2014) evaluated the *in vitro* digestibility of N in several protein sources and observed that adult house cricket (*Acheta domesticus*) had an N digestibility of 91.7%, whereas PM was lower at 87.9%. In the same study, the AA score, intended to represent the nutritional quality of a protein source, was higher for house cricket than for PM when using the National Research Council’s Nutrient Requirements of Dogs and Cats (NRC 2006) minimal requirements for growth of puppies as the reference, suggesting that cricket had a more balanced AA profile. The greater digestibility of those AA could be a major factor affecting their bioavailability (Gaudichon and Calvez 2021), supporting the greater postprandial plasma appearance of BCAA in dogs fed CKP-D. However, Kilburn et al. (2020) did not report a similar response when assessing the ATTD of CP in dogs fed diets containing CKP derived from *Gryllodes sigillatus* harvested at 5 to 6 wk of age. In that study, ATTD of CP decreased linearly as dietary CKP inclusion increased, at the expense of chicken meal, from 0% to 24%. The conflicting results between our study and that of Kilburn et al. (2020) may be explained by differences in diet composition, specifically TDF content. In the study by Kilburn et al. (2020), greater dietary inclusion of CKP led to higher TDF levels in the diets. However, in the current study, diets had more similar TDF content (10% TDF on a DMB in CTRL vs. 11% TDF on a DMB in CKP-D). Therefore, while the data from Kilburn et al. (2020) suggest that cricket may lower protein digestibility when included as a major protein source, possibly due to its chitin content, the findings from our study suggest that the postprandial AA bioavailability of the CKP-based diet was even greater than that of a diet with PM as the major protein source. Additionally, as discussed above, ATTD has major limitations when used to assess CP digestibility and cannot be extrapolated to AA utilization. It is also important to note that dietary CP in our study was estimated using the standard nitrogen-to-protein conversion factor of 6.25, assuming that protein contains 16% nitrogen. This factor was applied to the CKP ingredient to balance total CP across experimental diets. However, the use of 6.25 likely overestimates the CP content of CKP because it contains chitin, a nitrogen-containing polysaccharide. Consequently, a more specific conversion factor of 5.25 has been proposed for cricket-based ingredients to account for non-protein nitrogen (Boulos et al. 2020). When applying the 5.25 factor, the estimated CP content of CKP-D is 31.90% DM rather than 35.5% DM, which more closely aligns with the estimated CP of CTRL (33.4% DM).

Because plasma AA bioavailability is being evaluated, it is more appropriate to consider the AA composition of the diets and the proportion contributed by PM and CKP rather than relying solely on CP content. Although most AA had similar compositions across diets, with comparable contributions from the primary protein sources, small numerical differences were present and should be considered when interpreting the results. Therefore, differences in postprandial plasma AA responses cannot be attributed exclusively to the inclusion of PM or CKP. While balancing each individual AA across diets would help isolate ingredient effects, doing so would substantially alter ingredient composition. Consequently, the observed physiological responses should be interpreted as the overall effect of the diet rather than solely as the effect of the protein source. Another key factor influencing AA bioavailability is the manufacturing process and specific processing conditions used to produce ingredients and complete diets. PM is obtained through rendering (Crosbie et al. 2024), and CKP is produced by roasting insects. In both cases, heat treatment is applied during ingredient preparation prior to inclusion in the diets. Processes that involve high-heat application can influence protein quality by leading to crosslinking, including Maillard reactions, racemization, and oxidation of sulfur AA, all of which can render AA unavailable to the body (Rooijen et al. 2013; Loveday 2023). For example, Oba et al. (2019) reported that rendered chicken meal had lower AA digestibility using the cecectomized rooster assay for most IAA and DAA than steamed chicken cooked with a less intense heat treatment. Similarly, Calvez et al. (2024) compared a minimally processed infant formula with a conventionally heat-treated formula and found that, although the DIAAS was comparable, the minimally processed formula resulted in a larger postprandial concentration of plasma total AA and IAA in rats. It is essential to consider that the distinct processing methods used to produce each ingredient may have influenced the observed results as much as the protein sources themselves. PMs are produced via rendering, a process involving high heat and mechanical separation to concentrate proteins. In contrast, CKP is generally produced by roasting, a convective drying method that is inherently less heat-intensive. Importantly, not all PM are produced using similar rendering conditions, and variation in those conditions can impact the final quality and directly influence AA bioavailability, For example, Johnson et al. (1998) evaluated digestibility of animal meals processed in different temperatures and observed decreased AA digestibility on those that were processed using higher temperatures. We hypothesize that the potentially higher thermal load experienced by the PM during rendering compared to the roasting of the CKP may also be impacting the plasma AA bioavailability observed in this study. However, because we do not have detailed information on the manufacturing processes of the CKP and PM used in this study and given that processing conditions can vary widely across commercial facilities, it is not possible to isolate the specific impact of ingredient processing on the outcomes observed.

Mechanisms that affect AA bioavailability as a result of heat application apply not only to ingredient manufacturing but also to pet food production, where high-temperature and pressure processes, such as extrusion, can impact AA availability. Chen et al. (2025) reported the extrusion processing conditions used to produce both the CTRL and CKP-D utilized in the current study. During extrusion of the experimental diets, the preconditioner and extruder barrel temperatures were kept constant for both diets, but the specific mechanical energy and die temperatures were lower for the CKP-D than for the CTRL. Because CKP contained 18.1% fat compared to 13.8% in PM, the authors suggested that increased lubrication in the extruder barrel may have lowered the specific mechanical energy input, and consequently, lowered die temperature due to less friction and heat generation, which would further reduce the damage in the AA (Chen et al. 2025). The experimental diets were not formulated to have similar fat levels; rather, the CTRL was formulated to have higher fat to achieve a more balanced CP content across diets. The additional fat observed in the final nutrient composition of the diets was applied during the enrobing step and thus would not have contributed to this lubricating effect.

Even though the lower specific mechanical energy applied during the production of CKP-D compared to CTRL may have impacted AA bioavailability, and thus, the plasma AA kinetics observed in the present study, Dai et al. (2022) avoided this confounding factor by using a non-heat-treated beverage as the matrix delivery of cricket and beef protein and still reported greater plasma BCAA and IAA in humans after cricket was consumed compared with beef protein. Those findings suggest that, although differences in extrusion conditions may influence plasma AA kinetics, differences in BCAA iAUC may also reflect intrinsic properties of the ingredient source. However, the authors did not report how the cricket and beef protein ingredients used in the study were produced, which can impact AA bioavailability, as discussed above. Different ingredient sources vary in their protein structure, organization, and nonprotein components, which can affect AA digestibility and protein quality (Matthews et al. 2025). Moreover, solubility, gastric coagulation, and even protein molecular size can also impact how they are digested and absorbed (Deglaire et al. 2009; Rubio and Clemente 2010; Loveday 2023). All those factors require further characterization and may help explain why CKP-D led to greater IAA availability than CTRL.

Although the factors contributing to the greater IAA concentrations and BCAA iAUC in dogs fed CKP-D cannot be fully isolated or mechanistically explained in the current study, these results likely reflect multiple confounded factors related to diet composition, ingredient processing, extrusion conditions, and chemical changes occurring during production of experimental diets. This response may have important implications for postprandial protein metabolism, as these AA provide substrate and activate signaling pathways that promote muscle protein synthesis (Boirie et al. 1997; Vary and Lynch 2007). Importantly, the potentially greater supply of bioavailable AA from CKP-D could have led to more AA remaining in circulation due to prolonged availability or reduced immediate utilization, but this may also be related to increased mobilization of AA from body protein reserves during fasting (Nørgaard et al. 2021) due to a potential limiting AA.

Most DAA, as well as their total, followed trends similar to those of the IAA, with greater concentrations observed in dogs fed CKP-D. In contrast, Tau and Gln were higher in dogs fed CTRL, with the latter showing no change in plasma concentration after meal consumption, unlike the other AA. Even though Gln concentrations did not change in the blood after consuming a diet, this AA was the one found at the highest concentration in the blood, in agreement with other studies (de Godoy et al. 2014; Azuma et al. 2016). The small intestine can utilize Gln from the arterial circulation, in which Gln may be synthesized from BCAA and α-ketoglutarate, or from the intestinal lumen (Wu 2009). Thus, a plausible explanation for the stable Gln concentrations over the 360 min postprandial period is that most dietary Gln is metabolized by enterocytes during first-pass metabolism (Wu 1998; Wu 2009) as a primary energy source for these cells. This mechanism was also proposed by Banton et al. (2025) in a study evaluating postprandial AA responses in dogs fed diets with varying CP levels. The rationale for the higher plasma Tau concentrations in dogs fed CTRL is less clear but may be attributed to greater dietary Tau availability in CTRL compared to CKP-D or to greater endogenous synthesis (Miyazaki 2024).

The present study has additional limitations than those mentioned previously that prevent all observed effects from being attributed solely to CKP. For instance, the macronutrient composition of diets differed, which could have affected digestibility and, consequently, AA kinetics. We also lacked detailed information on the manufacturing conditions of the specific PM and CKP batches used in this study, and processing parameters can vary widely between facilities, potentially affecting protein quality. Although future studies would benefit from feeding PM and CKP as the sole protein source in a postprandial test to more clearly isolate ingredient effects, such an approach would not capture the impact of processing conditions within a complex food matrix, such as extruded pet food. Despite these limitations, the current findings highlight the enhanced AA bioavailability associated with CKP-D and underscore the need for further research to elucidate the mechanisms underlying these responses.

## Conclusion

Replacing 30% PM with CKP in extruded dog diets did not affect glucose or insulin kinetics, with the exception of a slightly greater glucose Cmax in dogs fed CKP-D. However, dogs fed CKP-D had greater postprandial availability of most AA, particularly BCAA, indicating greater potential to stimulate protein synthesis. Further research is required to isolate the individual effects of CKP and PM from the confounding effects of ingredient manufacturing conditions, food matrix changes, and pet food production to better understand the potential mechanisms underlying the greater postprandial bioavailability of AA in dogs fed CKP-D compared to those fed CTRL.

## Supplementary Material

skag162_Supplementary_Data

## Abbreviations

AA: amino acid
ATTD: apparent total tract digestibility
BCAA: branched chain amino acid
CKP: cricket powder
CKP-D: cricket powder diet
Cmax: maximum concentration
CP: crude protein
CTRL: control diet
DAA: dispensable amino acid
DIAAS: digestible indispensable amino acid score
DM: dry matter
HOMA-IR: Homeostatic model assessment for insulin resistance
iAUC: incremental area under the curve
IAA: indispensable amino acid
ME: metabolizable energy
NetAUC: net area under the curve
PM: poultry meal
Tmax: time to maximum concentration
