# Supplementary material for: Postprandial plasma amino acid, glucose, and insulin responses in healthy dogs following the consumption of extruded diets containing cricket powder or poultry meal
Source: J Anim Sci. 2026 May 22;104:skag162. doi: 10.1093/jas/skag162 (PMC13360706; doi:10.1093/jas/skag162)
Supplement: skag162_Supplementary_Data [file skag162_supplementary_data.docx]

**Supplementary Table 1**. Effect of dietary treatment on plasma glucose and insulin concentrations^1^ over time in dogs

|  | Glucose, mmol/L | | Insulin, mU/L | |
| --- | --- | --- | --- | --- |
| Time (min) | CTRL^2^ | CKP-D | CTRL | CKP-D |
| 0 | 5.56^e^ (0.17) | 5.61^e^ (0.17) | 6.43^hi^ (1.41) | 5.37^hi^ (1.18) |
| 5 | 5.80^cd^ (0.18) | 5.96^cd^ (0.20) | 11.7^efg^ (2.74) | 9.64^efg^ (2.43) |
| 10 | 5.46^cde^ (0.20) | 5.98^cde^ (0.20) | 4.81^hi^ (1.21) | 7.37^hi^ (1.86) |
| 15 | 5.84^cde^ (0.17) | 5.79^cde^ (0.18) | 6.77^gh^ (1.58) | 8.11^gh^ (1.90) |
| 30 | 5.75^cde^ (0.17) | 5.90^cde^ (0.17) | 10.2^ef^ (2.25) | 12.6^ef^ (2.77) |
| 60 | 6.07^ab^ (0.17) | 6.45^ab^ (0.18) | 19.2^abc^ (4.23) | 27.3^abc^ (6.41) |
| 120 | 6.43^a^ (0.18) | 6.59^a^ (0.18) | 25.0^ab^ (5.50) | 31.3^ab^ (7.35) |
| 180 | 6.26^a^ (0.17) | 6.34^a^ (0.18) | 32.1^a^ (7.05) | 31.5^a^ (7.41) |
| 240 | 6.05^cd^ (0.17) | 5.84^cd^ (0.18) | 23.8^bc^ (5.23) | 17.9^bc^ (4.20) |
| 300 | 6.00^c^ (0.17) | 5.93^c^ (0.18) | 21.4^cd^ (4.71) | 13.7^cd^ (3.22) |
| 360 | 6.01^bc^ (0.17) | 6.00^bc^ (0.18) | 15.4^de^ (3.39) | 10.5^de^ (2.46) |
| 420 | 5.87^cd^ (0.17) | 5.91^cd^ (0.18) | 6.50^fgh^ (1.43) | 9.08^fgh^ (2.14) |
| 480 | 5.85^cde^ (0.18) | 5.63^cde^ (0.18) | 6.04^hi^ (1.41) | 5.20^hi^ (1.22) |
| 540 | 5.97^cde^ (0.18) | 5.64^cde^ (0.18) | 7.84^hi^ (1.84) | 5.14^hi^ (1.21) |
| 600 | 5.79^cd^ (0.18) | 5.97^cd^ (0.20) | 4.75^i^ (1.11) | 4.67^i^ (1.10) |
| 660 | 5.74^de^ (0.18) | 5.60^de^ (0.18) | 3.25^i^ (0.82) | 6.23^i^ (1.46) |
| 720 | 6.02^cd^ (0.18) | 5.81^cd^ (0.18) | 4.53^i^ (1.06) | 4.86^i^ (1.14) |
| *P*-Trt | 0.837 | | 0.961 | |
| *P*-Time | <0.001 | | <0.001 | |
| *P*-Interaction | 0.422 | | 0.345 | |
| ^a-i^Means within the same column lacking the same letter differ significantly with respect to time effect at *P* < 0.05.  ^1^Means (SEM; Standard error of the mean).  ^2^CTRL, control diet; CKP-D, cricket powder diet. | | | | |

**Supplementary Table 2**. Standard error of means for plasma indispensable amino acid concentrations.

| Trait^1^ | Treatment^2^ | Time, min | | | | | | | | |
| --- | --- | --- | --- | --- | --- | --- | --- | --- | --- | --- |
|  |  | 0 | 30 | 60 | 90 | 120 | 180 | 240 | 300 | 360 |
| Arg | CTRL | 11 | 11 | 11 | 11 | 11 | 11 | 12 | 11 | 11 |
|  | CKP-D | 12 | 12 | 12 | 12 | 12 | 12 | 12 | 12 | 12 |
| His | CTRL | 5 | 5 | 5 | 5 | 5 | 5 | 5 | 5 | 5 |
|  | CKP-D | 6 | 6 | 5 | 5 | 5 | 5 | 6 | 5 | 5 |
| Ile | CTRL | 9 | 9 | 9 | 9 | 9 | 9 | 9 | 9 | 9 |
|  | CKP-D | 10 | 10 | 9 | 9 | 10 | 9 | 10 | 9 | 9 |
| Leu | CTRL | 21 | 21 | 21 | 21 | 21 | 21 | 21 | 21 | 21 |
|  | CKP-D | 23 | 22 | 22 | 22 | 22 | 22 | 22 | 22 | 22 |
| Lys | CTRL | 11 | 11 | 11 | 11 | 11 | 11 | 12 | 11 | 11 |
|  | CKP-D | 13 | 12 | 12 | 12 | 12 | 12 | 12 | 12 | 12 |
| Met | CTRL | 6 | 6 | 6 | 6 | 6 | 6 | 6 | 6 | 6 |
|  | CKP-D | 7 | 7 | 7 | 7 | 7 | 7 | 7 | 6 | 6 |
| Phe | CTRL | 3 | 3 | 3 | 3 | 3 | 3 | 3 | 3 | 3 |
|  | CKP-D | 4 | 3 | 3 | 3 | 3 | 3 | 3 | 3 | 4 |
| Thr | CTRL | 31 | 31 | 31 | 31 | 31 | 31 | 32 | 31 | 31 |
|  | CKP-D | 32 | 32 | 32 | 32 | 32 | 32 | 32 | 32 | 32 |
| Trp | CTRL | 6 | 6 | 6 | 6 | 6 | 6 | 6 | 6 | 6 |
|  | CKP-D | 6 | 6 | 6 | 6 | 6 | 6 | 6 | 6 | 6 |
| Val | CTRL | 17 | 17 | 17 | 17 | 17 | 17 | 18 | 17 | 17 |
|  | CKP-D | 19 | 19 | 18 | 19 | 18 | 18 | 19 | 18 | 18 |
| Total AA | CTRL | 185 | 185 | 185 | 185 | 185 | 185 | 192 | 185 | 185 |
|  | CKP-D | 205 | 205 | 194 | 194 | 194 | 194 | 205 | 194 | 194 |
| BCAA | CTRL | 47 | 47 | 47 | 47 | 47 | 47 | 48 | 47 | 47 |
|  | CKP-D | 50 | 50 | 49 | 50 | 49 | 49 | 50 | 49 | 49 |
| IAA | CTRL | 101 | 101 | 101 | 101 | 101 | 101 | 104 | 101 | 101 |
|  | CKP-D | 109 | 105 | 105 | 105 | 105 | 105 | 109 | 105 | 105 |
| ^1^AA, amino acid; BCAA, branched chain amino acid, IAA, indispensable amino acid  ^2^CTRL, control diet; CKP-D, cricket powder diet. | | | | | | | | | | |

**Supplementary Table 3**. Standard error of means for plasma dispensable amino acid concentrations.

| Trait^1^ | Treatment^2^ | Time, min | | | | | | | | |  |
| --- | --- | --- | --- | --- | --- | --- | --- | --- | --- | --- | --- |
|  |  | 0 | 30 | 60 | 90 | 120 | 180 | 240 | 300 | 360 |  |
| Ala | | CTRL | 24 | 24 | 24 | 24 | 24 | 24 | 25 | 24 | 24 |
|  |  | CKP-D | 27 | 27 | 27 | 26 | 26 | 26 | 27 | 26 | 26 |
| Asn | | CTRL | 5 | 5 | 5 | 5 | 5 | 5 | 6 | 5 | 5 |
|  |  | CKP-D | 6 | 6 | 6 | 6 | 6 | 6 | 6 | 6 | 6 |
| Gln | | CTRL | 45 | 45 | 45 | 45 | 45 | 45 | 45 | 45 | 45 |
|  |  | CKP-D | 47 | 47 | 46 | 46 | 46 | 46 | 47 | 46 | 46 |
| Glu | | CTRL | 6 | 6 | 6 | 6 | 6 | 6 | 6 | 6 | 6 |
|  |  | CKP-D | 7 | 7 | 7 | 7 | 7 | 7 | 7 | 7 | 7 |
| Gly | | CTRL | 14 | 14 | 14 | 14 | 14 | 14 | 14 | 14 | 14 |
|  |  | CKP-D | 16 | 16 | 16 | 16 | 16 | 16 | 16 | 16 | 16 |
| Orn | | CTRL | 2 | 2 | 2 | 2 | 2 | 2 | 2 | 2 | 2 |
|  |  | CKP-D | 3 | 3 | 3 | 3 | 3 | 3 | 3 | 3 | 3 |
| Pro | | CTRL | 21 | 21 | 21 | 21 | 21 | 21 | 22 | 21 | 21 |
|  |  | CKP-D | 23 | 23 | 23 | 22 | 22 | 22 | 22 | 22 | 22 |
| Ser | | CTRL | 13 | 13 | 13 | 13 | 13 | 13 | 13 | 13 | 13 |
|  |  | CKP-D | 14 | 14 | 13 | 13 | 13 | 14 | 14 | 13 | 13 |
| Tau | | CTRL | 8 | 8 | 8 | 8 | 8 | 8 | 8 | 8 | 8 |
|  |  | CKP-D | 9 | 9 | 8 | 8 | 8 | 8 | 9 | 8 | 8 |
| Tyr | | CTRL | 5 | 5 | 5 | 5 | 5 | 5 | 5 | 5 | 5 |
|  |  | CKP-D | 5 | 5 | 5 | 5 | 5 | 5 | 5 | 5 | 5 |
| DAA | | CTRL | 98 | 98 | 98 | 98 | 98 | 98 | 102 | 98 | 98 |
|  |  | CKP-D | 110 | 109 | 109 | 104 | 104 | 104 | 109 | 104 | 104 |
| ^1^DAA, dispensable amino acid  ^2^CTRL, control diet; CKP-D, cricket powder diet. | | | | | | | | | | | |
